# Pb^2+^ recovery from real water samples by adsorption onto nano Fe_3_O_4_/chitosan‐acrylamide hydrogel ions in real water samples

**DOI:** 10.1049/nbt2.12126

**Published:** 2023-03-21

**Authors:** Arman Samadzadeh Mamaghani, Mohammadreza Manafi, Mohammad Hojjati

**Affiliations:** ^1^ Faculty of Science Department of Applied Chemistry South Tehran Branch Islamic Azad University Tehran Iran

**Keywords:** adsorption, materials preparation, sorption

## Abstract

This study examined the removal of Pb(II) using magnetic chitosan hydrogel adsorbent from diverse sample waters. Spectrometry was used to track the effects of magnetic acrylamide nanocomposite dose, pH extraction, and contact duration on Pb(II) removal from sample water. This research also looked at adsorption isotherm models for the sorption of Pb(II). The magnetic chitosan hydrogel adsorbent Pb(II) adsorption capability was 31.74 mg/g respectively. The Freundlich isotherm model fits the removal of Pb(II) utilising magnetic chitosan hydrogel adsorbent. In addition, this adsorbent was shown to have a *q*_max_ value of 31.74 mg/g of Pb^2+^ ions, which is considered to be of high efficiency for Pb^2+^ ion removal. The studied kinetic models have determined that the pseudo‐second‐order linear model is more suitable to explain the adsorption of lead (II) on magnetic chitosan hydrogel adsorbent. Also, chemical adsorption is the rate‐limiting step in the adsorption process of lead (II) ions.

## Graphical abstract

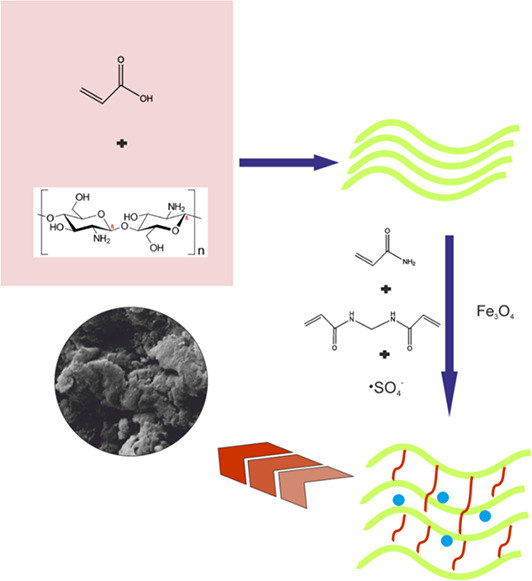

## 1 INTRODUCTION

Today, in many regions of the world, heavy metals and dyeing wastewater are the primary water resource contaminants. These pollutants wastewater has been produced in significant quantities by industrial activity. Wastewater of heavy metals has the potential to harm an organism's genetics. Toxic compounds may now be dealt with using a variety of techniques, including membrane separation, ion exchange, chemical deposition, electrochemical removal, adsorption, coagulation, flocculation, and more [1, 2, 3]. The adsorption technique is better than other methods because it is inexpensive, has an easy design, and is very effective. It is extensively used since it is straightforward and highly effective. Traditional adsorbents, however, struggle to reduce harmful chemicals in wastewater to a safe level. The most recent adsorbents to be employed in recent years to remediate pollutants are hydrogels. They are simple to use and reuse and have outstanding mechanical qualities [4, 5, 6, 7, 8, 9, 10].

Heavy metal ions from industrial wastewater, such as Cu(II), Pb(II), Mg(II), Hg(II), and Cd(II), are a significant source of groundwater contamination. These ions should be removed from wastewater before disposal since they are hazardous to both humans and aquatic life. Numerous hydrogels, including cellulose grafted acrylic acid (C‐g‐AA), chitosan hydrogel with 2,5‐dimercapto‐1,3,4‐thiodiazole (CTS‐DMTD), PVA hydrogel biomass of Penicillium cyclopium, and starch grafted acrylic acid/montmorillonite (S‐g‐AA/MMT), have been used in biosynthesis processes and as adsorbents [1, 2, 11, 12].

The three‐dimensional network structure of hydrogels allows for the retention of water inside the network structure. Hydrophilic or hydrophobic hydrogels can be made using polar or non‐polar monomers. Prepared hydrophobic hydrogels for certain purposes. Initiators, cross‐linkers, and monomers are typically involved.

Polar monomers are in situ polymerised to create hydrophilic hydrogels. For the creation of hydrogels, polymerisation processes, such as bulk, emulsion, and solution polymerisation are employed [13, 14].

As a result, the hydrogel has a unique place in the wastewater treatment industry. Graphene oxide, inorganic clay, cellulose, acrylic acid, and synthetic polymers are now the primary components of synthetic hydrogels. Because of its special compatibility, chitosan is extensively found in nature and employed in the medical industry. Additionally, chitosan is employed as an adsorbent because it includes a lot of active groups that may bind, coordinate, and remove ions with hazardous compounds in the medium [15, 16, 17, 18].

The polyacrylic acid hydrogel was used to remove Cu(II) and Ni(II) ions from water [3]. The 2‐acrylamido‐2‐methyl‐1‐propane sulphonic acid‐based magnetic responsive hydrogels were used to remove heavy metal Cd(II), Co(II), Fe(III), Pb(II), Cr(III), Ni(II), and Cu(II) ions [19]. A hydrogel made of magnetic vinyl pyridine was used to extract the metal ions U and Th from aqueous settings [20].

The produced hydrogel for the removal of the Pb(II) ion, which was reinforced with multi‐walled carbon nanotubes, was reused for four cycles, with an adsorption effectiveness of more than 80% after four cycles [21]. It has been reported that hydrogels made of poly (acrylic acid‐co‐hydroxyethyl methacrylate) act as selective Pb(II), Cu(II), and Zn(II) ion adsorbents [22].

In this work, a hydrogel adsorbent was created using chitosan and acrylamide/acrylic acid/Fe_3_O_4_. The amount of Pb(II) ions adsorption was tested in different conditions. Kinetic models and adsorption isotherms for the adsorption of lead ions in an aqueous medium were investigated.

## 2 MATERIALS AND METHODS

### 2.1 Materials

Acrylamide (98.0%), acrylic acid (98.0%), N,N′‐methylenebisacrylamide (97.0%), and chitosan with a deacetylation degree of 96% were purchased from Sigma‐Aldrich. Merck supplied the ammonium persulfate, FeCl_3_•6H_2_O, and FeCl_2_•H_2_O (Darmstadt Germany). Pb (NO_3_)_2_ dissolved in deionised water to make the stock of Pb^2+^ solutions (300 mg/L). Analytical grade chemicals were employed throughout the investigation, and deionised water was used in every experiment.

### 2.2 Characterisations

A thermogravimetric analyser TGA2 (Mettler Toledo) is used to examine the hydrogel's heat stability. Samples are also analysed using a Fourier infrared spectrometer (Perkin Elmer). Test adsorption of the lead ions concentration using the graphite furnace atomic spectrometer (Agilent); X‐ray patterns analysis was carried out with Pert Pro X (Panalytical) The FESEM was used to capture the photos (ZEISS, Sigma VP). A VSM AGFM/VSM3886 device was used to conduct magnetisation measurements (Meghnatis Daghigh Kavir Company). Magnetic separation was accomplished using a super magnet with a 1.2 T magnetic field (N35 model from Tehran Magnet).

### 2.3 Synthesis of nano iron oxide

Separately, 2.5 g of FeCl_2_ and 5 g of FeCl_3_ were dissolved in 50 mL of distilled water. A flask was used to combine the two solutions. One side of the mixture was kept continually under nitrogen gas, while the other side received 50 mL of 3 M NaOH, which was added and vigorously stirred for 1 h at 60°C. It was then rinsed in distilled water to get the pH level down to 7, and finally, it was dried in a 70°C oven.

### 2.4 Hydrogel preparation

**SCHEME 1:**
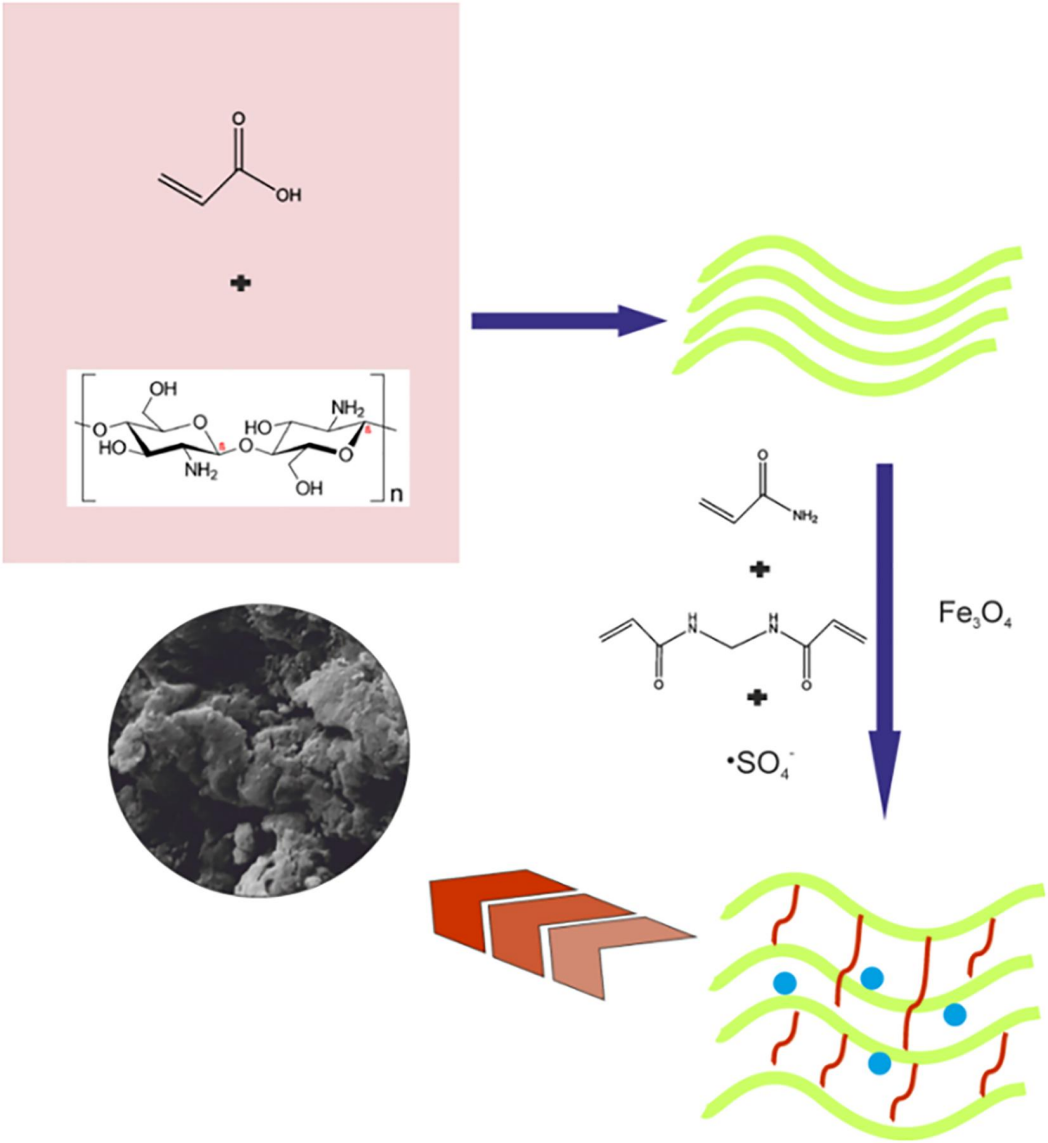
Synthesis of magnetic chitosan hydrogel adsorbent.

The 20 mL of distilled water is used to dissolve 5 mL of acrylic acid monomer, and then 0.1 g of chitosan is added. The mixture is then well stirred for 4 h before being strained. A 5 g of acrylamide was dissolved in 20 mL of distilled water before adding acrylic acid/chitosan (filtered solution). The monomer solution was added to the 0.1 g of nano iron oxide that had been dissolved in 10 mL of water in a small beaker, stirred with a glass stirrer, and then 0.2 g of ammonium persulfate and 0.05 g of N,N′‐methylenebisacrylamide were combined in the same beaker and placed at a temperature of 70°C under nitrogen gas, where polymerisation took place. The polymer was then dried in a vacuum oven at a temperature of 60°C (Scheme 1.).

### 2.5 Sorption experiments

This study used lead (II) heavy metal ions to conduct a series of adsorption studies to test the efficacy of the hydrogel materials for contaminants. The concentration of the lead ions was measured  using the graphite furnace atomic spectrometer. In addition, 200 mL of 30 and 50 μg/L of Pb^2+^ ion solution was prepared. Equation (1) was used to represent the lead (II) adsorption capacity (*q*_*e*_) for ions as follows [23]:

(1) $$q_{e}=\frac{\left(C_{0}-C_{e}\right)V}{m}$$

where *V* is the volume of the solution (L), *m* is the weight of the adsorbent, and *C*_0_ and *C*_*e*_ are the beginning and final concentrations (mg/L) of Pb (II) ions.

The pH value of the solution containing the Pb (II) ions was changed to range from 2 to 7 using either HCl (1 M) or NaOH (1 M) in order to evaluate the impact of pH on the effectiveness of adsorption. The ammonium buffer solution was used in alkaline pH to prevent lead ion precipitation. The experiment related to all adsorption parameters was repeated three times.

## 3 RESULTS AND DISCUSSION

### 3.1 Characterisations

#### 3.1.1 FTIR analysis

**FIGURE 1:**
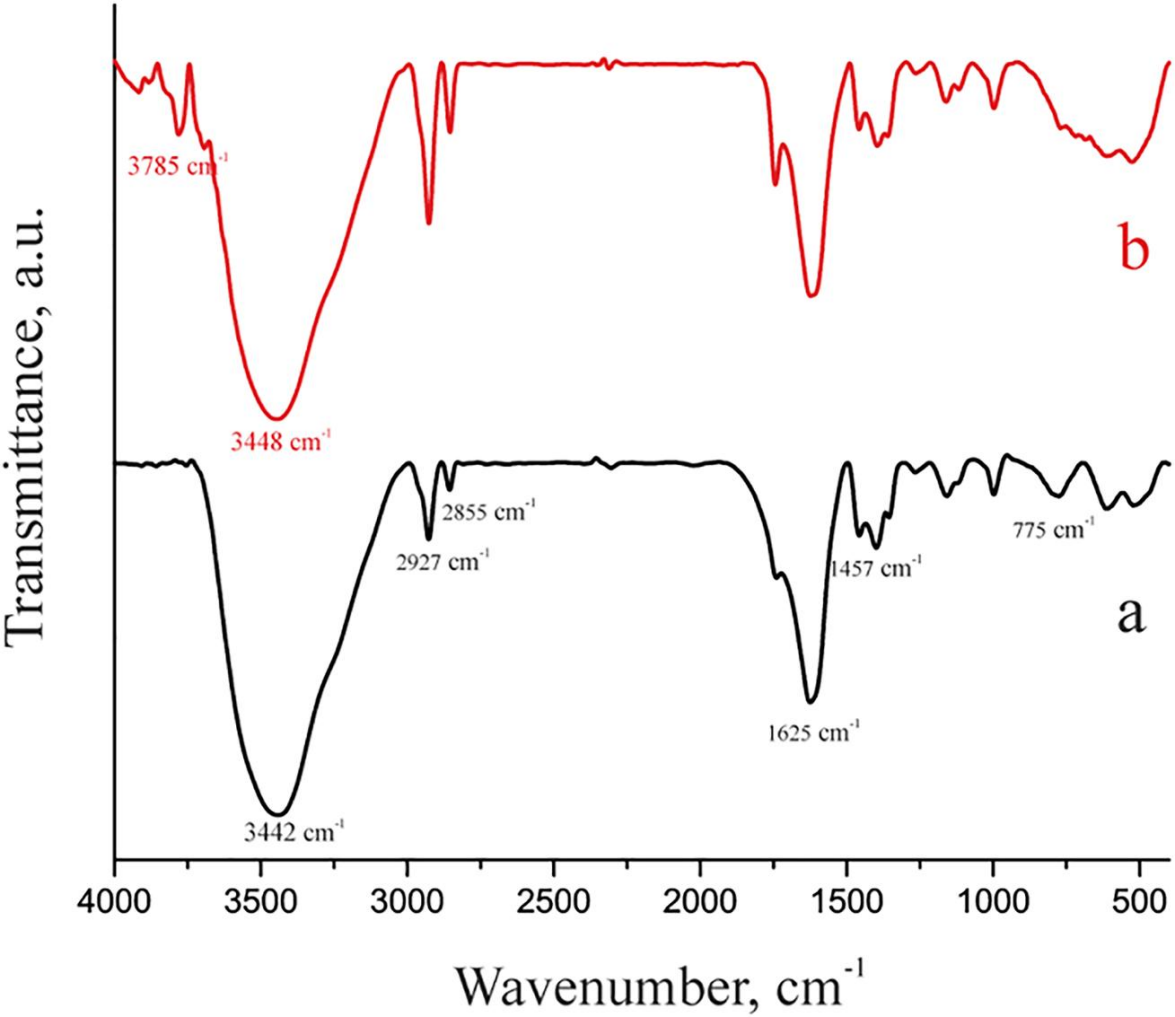
Fourier transform infrared spectra of magnetic chitosan hydrogel adsorbent (a) before Pb(II) adsorption and (b) after Pb(II) adsorption.

Figure 1 presents the findings of the fourier transform infrared (FTIR) investigation of the magnetic chitosan hydrogel adsorbent (Figure 1a). The peak at 3442 cm^−1^ in the FTIR spectra is related to the OH. The ‐CH stretch bond is related to the peaks at 2927 cm^−1^ and 2855 cm^−1^ [24, 25]. Additionally, the stretching C=O vibration reached its peak at 1625 cm^−1^. Bending vibration connected the Fe‐O band in Fe_3_O_4_ to the peak at 775 cm^−1^ [26]. The N‐H group peak at 1457 cm^−1^ illustrates the amidation reaction between the carboxyl groups of polyacrylic acid and the amine groups of chitosan [27]. The FTIR spectra after Pb(II) adsorption is shown in Figure 1b. When Pb(II) was added, the broad peak of the adsorbent that was attributed to NH or OH at 3442 cm^−1^ was altered to 3446 and 3780 cm^−1^, proving that NH or OH may interact with Pb(II) ions.

#### 3.1.2 X‐ray diffraction analysis

**FIGURE 2:**
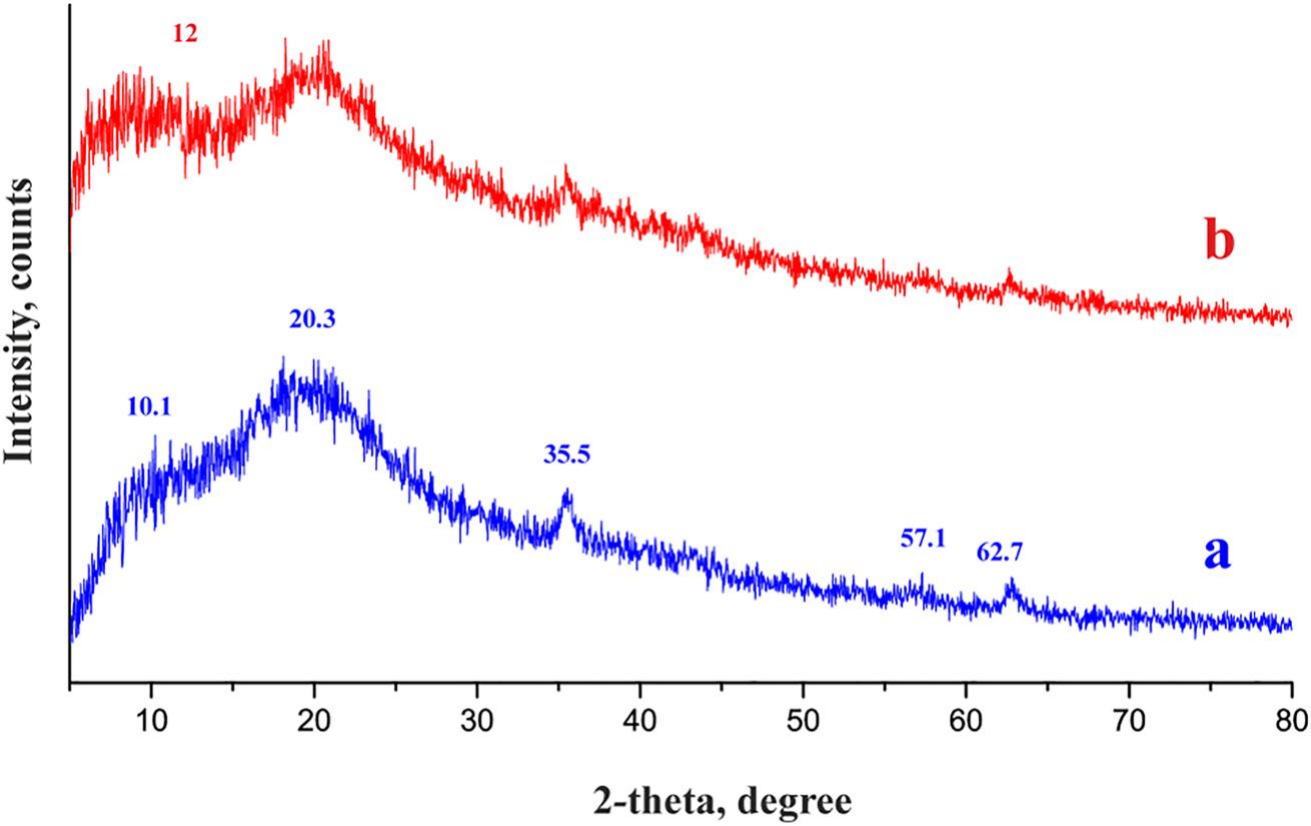
XRD pattern of magnetic chitosan hydrogel adsorbent (a) before Pb(II) adsorption and (b) after Pb(II) adsorption.

Only two significant peaks, positioned at 8° and 21°, in the magnetic chitosan hydrogel adsorbent XRD pattern demonstrate acrylamide's amorphous nature. The presence of chitosan is shown by a peak at 2*θ* = 10° in Figure 2a [28]. Peaks at 2*θ* = 35.5°, 57.15°, and 62.77°, respectively, correlate to magnetic particles and support the presence of Fe_3_O_4_ particles [6, 29].

#### 3.1.3 SEM analysis

According to the scanning electron microscopy (SEM) findings in Figure 3a, the magnetic chitosan hydrogel absorbent is made with nanometre particles that are placed on it and can be related to iron nanoparticles. Also, after the adsorption of Pb^2+^ ions by the magnetic chitosan hydrogel, it can be seen that the morphology of the adsorbent has changed Figure 3b. The energy dispersive spectroscopy (EDS) analysis showed the constituent components of the adsorbent (Figure 3a), The EDS analysis showed the constituent components of the adsorbent (Figure 3a), and also EDS represented the Pb^2+^ ions adsorption was successfully by the magnetic chitosan hydrogel absorbent (Figure 3b).

FIGURE 3(a) Scanning electron microscopy (SEM) image of magnetic acrylamide nanocomposite before Pb^2+^ ions sorption. (b) SEM image of magnetic acrylamide nanocomposite after Pb^2+^ ions sorption.

**Figure:**
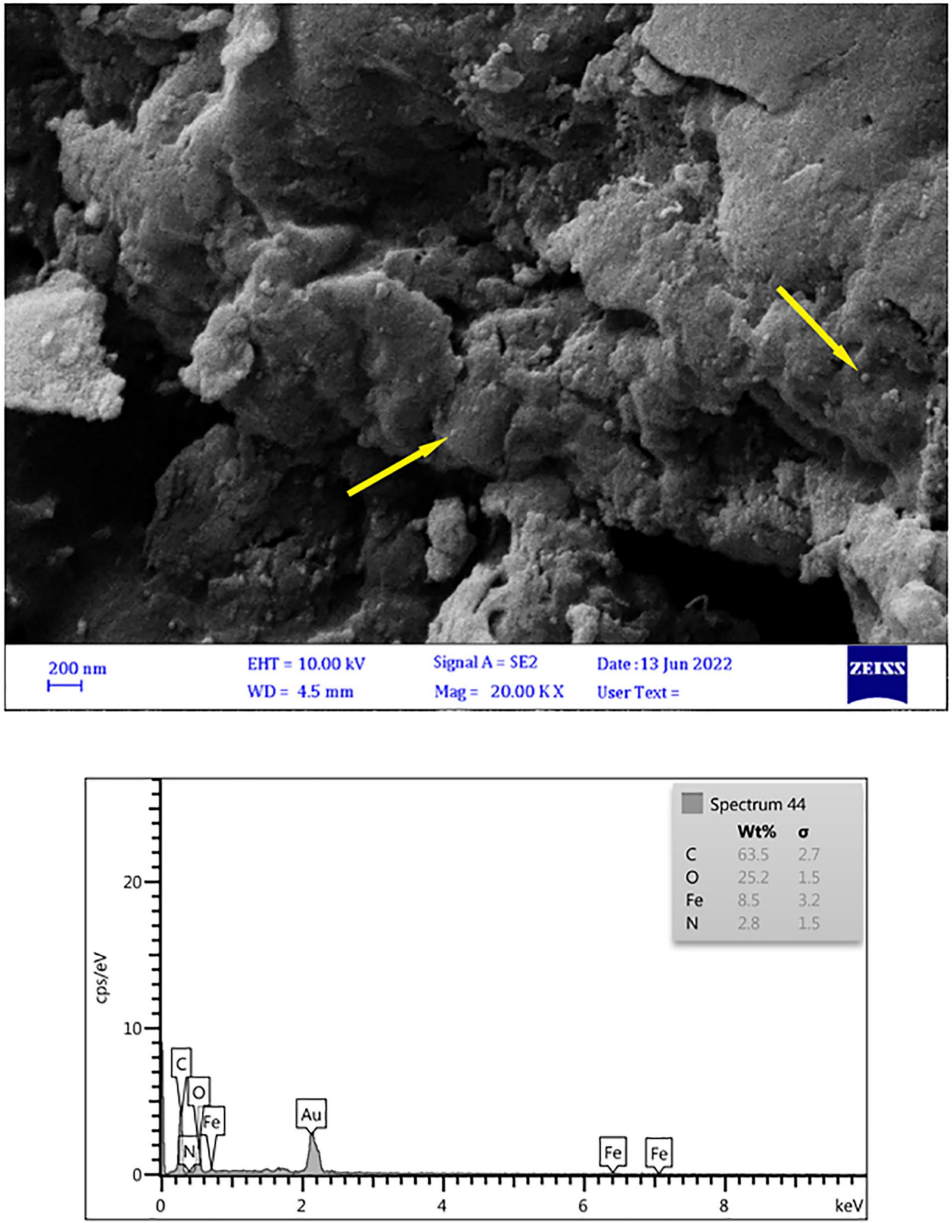

**Figure:**
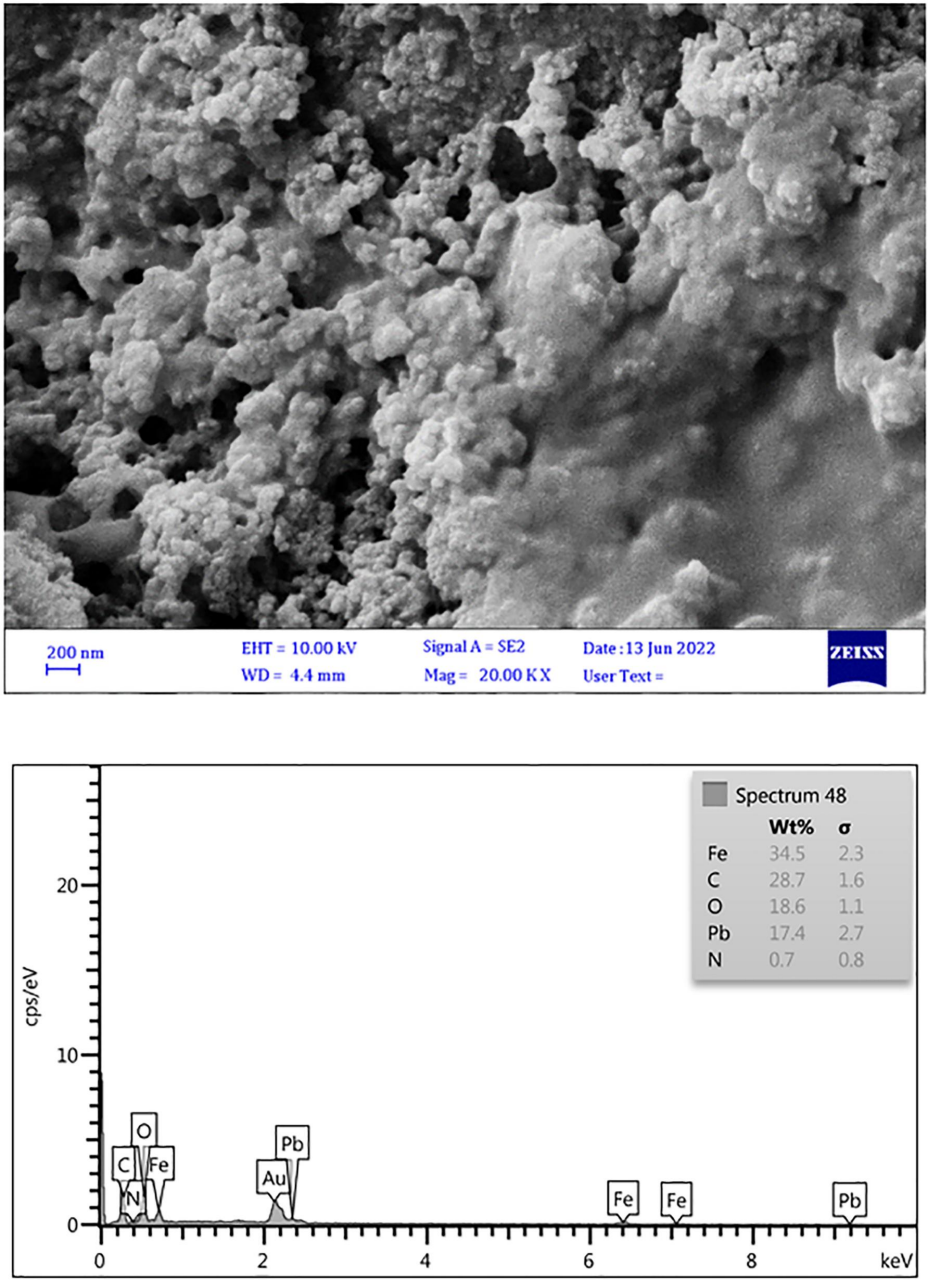

#### 3.1.4 Thermal gravimetric analysis and Differential thermogravimetry analysis

**FIGURE 4:**
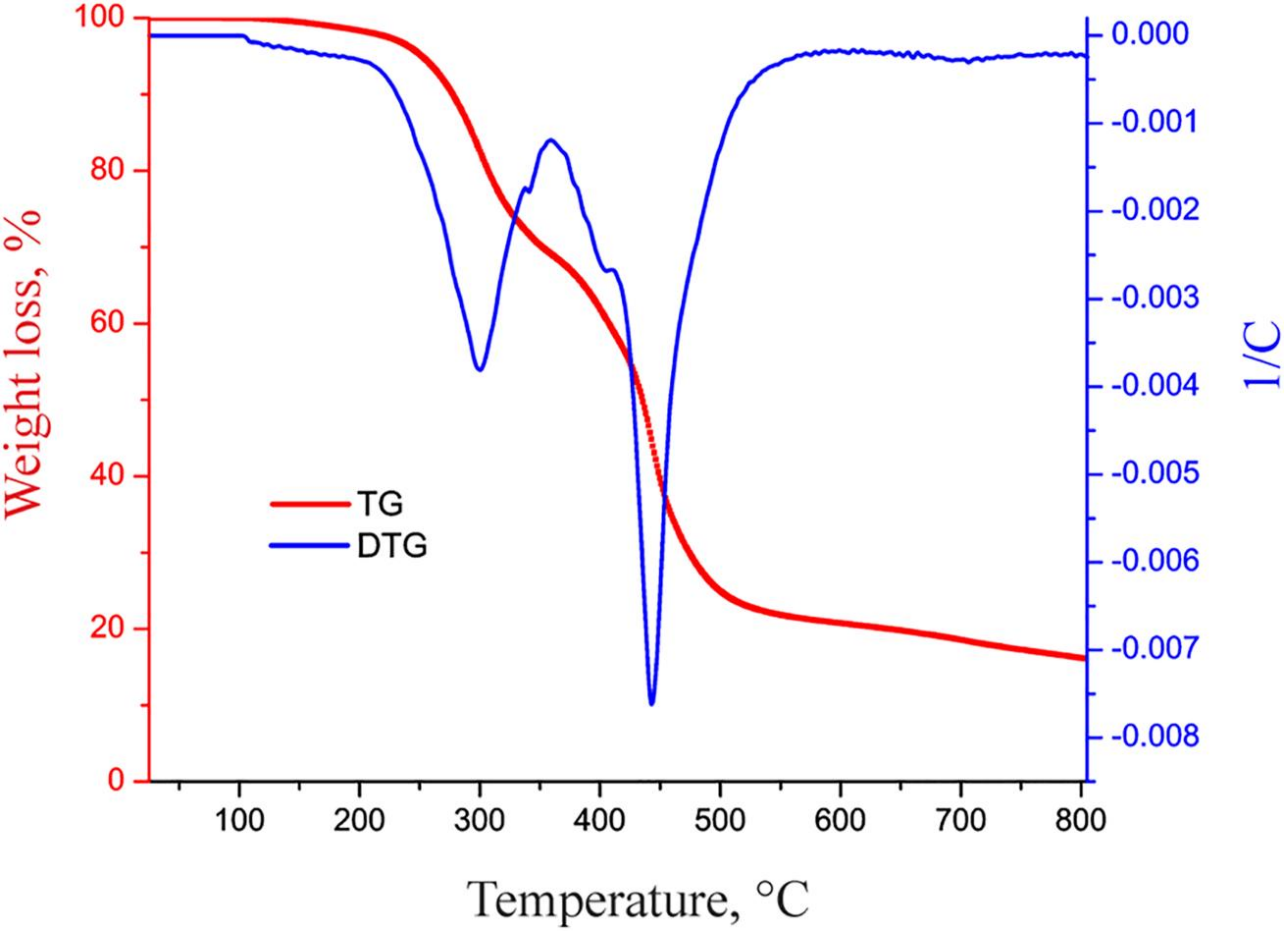
Thermal gravimetric analysis and differential thermogravimetry curves of magnetic chitosan hydrogel adsorbent.

According to the curves of the magnetic chitosan hydrogel adsorbent (Figure 4), the evaporation of any remaining moisture in the hydrogel structure is what causes the magnetic hydrogel structure's initial stage of weight loss to take place at a temperature below 100°C. It was discovered that weight loss occurred between 225 and 350°C, and this was caused by the breakdown of acrylic acid and acrylamide. The following weight loss was caused by the breakdown of chitosan in the range of 400°C.

#### 3.1.5 Magnetic field analysis

**FIGURE 5:**
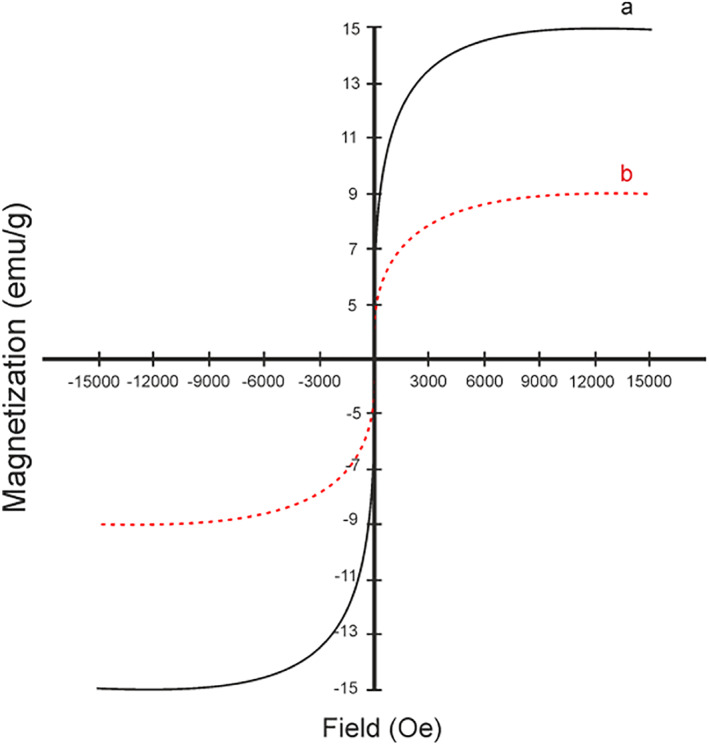
(a) VSM magnetisation curve of Fe_3_O_4_ nanoparticles and (b) magnetic chitosan hydrogel adsorbent.

The magnetic chitosan hydrogel adsorbent received the magnetic field. The magnetic properties of the Fe_3_O_4_ nanoparticles and the magnetic chitosan hydrogel adsorbent are 15 and 9 emu/g respectively. The decline from 15 to 9 emu/g was caused by the strong chemical interactions that were created between the Fe_3_O_4_ particles and the functional groups of the magnetic hydrogel (Figure 5).

### 3.2 pH optimisation

**FIGURE 6:**
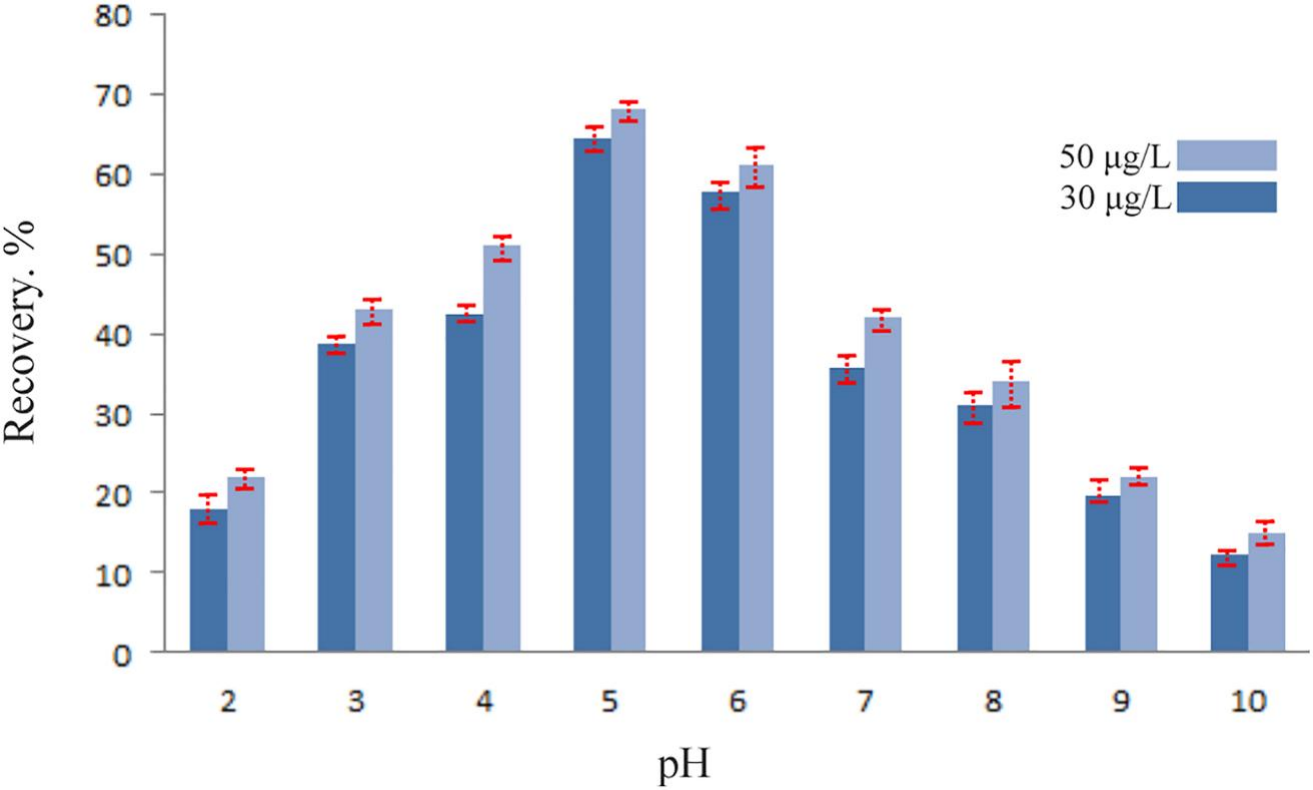
The adsorption of Pb^2+^ ions with adsorbent in pH factor for 30 and 50 μg/L (0.02 g of adsorbent for 10 min).

The pH has a significant influence on the extraction of Pb^2+^ ions utilising magnetic chitosan hydrogel adsorbent. The effect of pH on the recovery of Pb^2+^ ions was evaluated at pH = 2–10. At pH values higher than 6, the extraction recovery falls off due to the precipitation of Pb^2+^ ions as hydroxides, as seen in Figure 6. The recovery was reduced at less than pH = 5 because the amine group of the chitosan in the nanocomposite was protonated and the H^+^ ions bound. As a result, pH = 5 generated a high contact between the Pb^2+^ ions and magnetic chitosan hydrogel adsorbent.

### 3.3 Amount of magnetic chitosan hydrogel adsorbent

**FIGURE 7:**
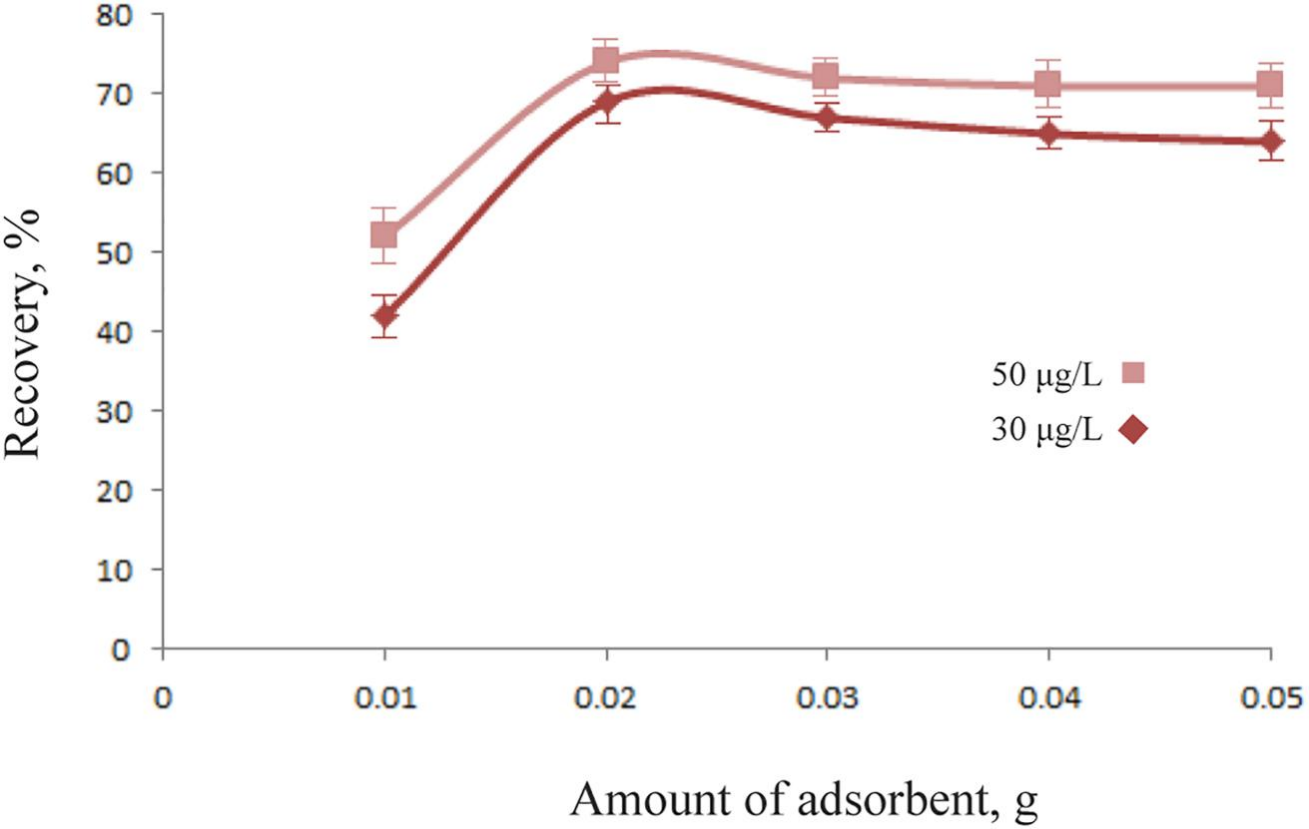
Effective of magnetic chitosan hydrogel adsorbent amount on Pb^2+^ ions sorption for 30 and 50 μg/L (pH = 5 for 10 min).

The best dose of magnetic chitosan hydrogel adsorbent was determined by testing several doses between 0.01 and 0.1 g (Figure 7). The optimal weight was 0.02 g, which had the maximum percentage adsorption value and recovery value. As a result, increasing the number of adsorbents enhances the surface sites for Pb^2+^ adsorption, increasing the efficacy of adsorption. With a reduced adsorbent dosage, the active sites available for ion adsorption are soon saturated, leading to a significant loss in adsorption efficiency.

### 3.4 Time effect

**FIGURE 8:**
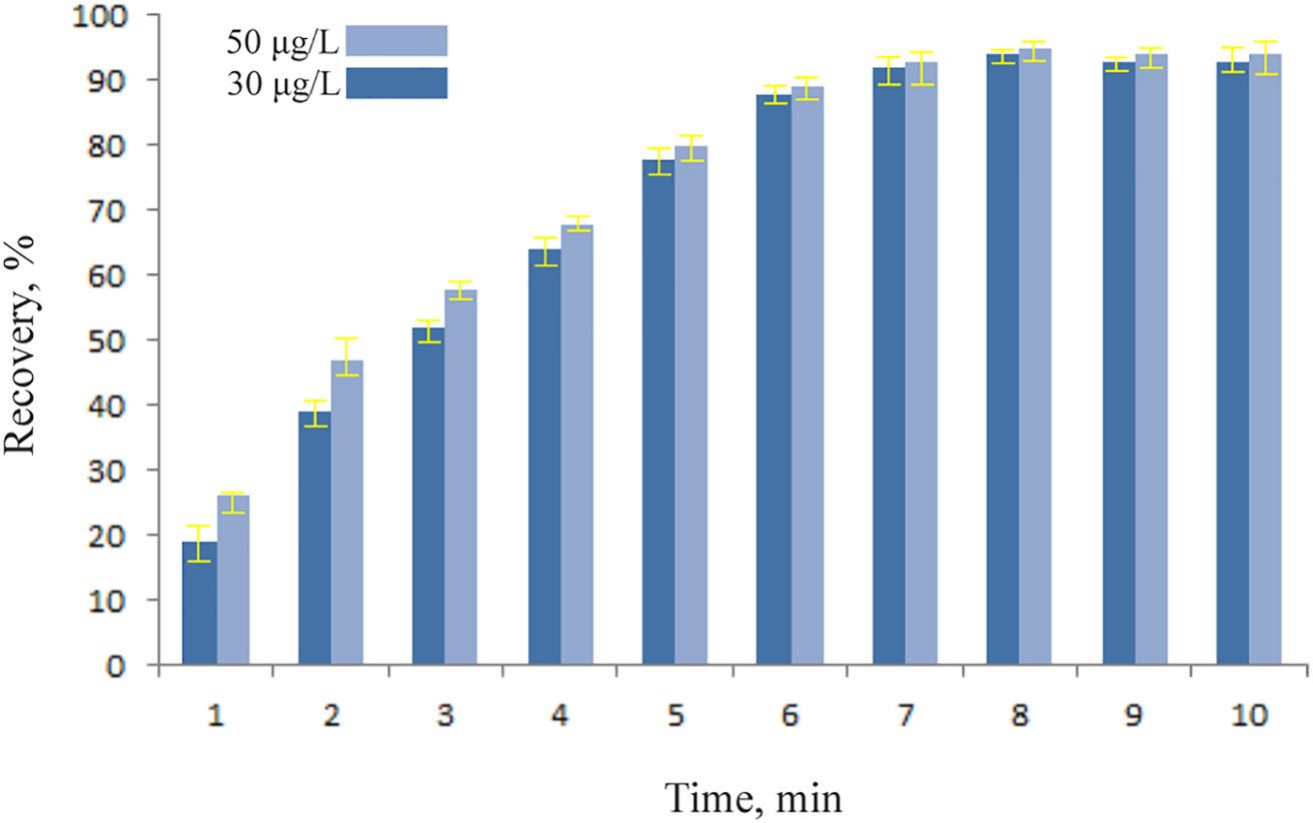
Effect of contact time on the Pb^2+^ ions adsorption with magnetic chitosan hydrogel for 30 and 50 μg/L (0.02 g of adsorbent in pH = 5).

For this, the recovery of Pb^2+^ ions at intervals of 1–10 min was investigated. The adsorption of the lead ions concentration using the graphite furnace atomic spectrometer. As shown in Figure 8, the rate of ion adsorption increases with time, and the amount is removed after 8 min. As the equilibrium between the Pb^2+^ ions and the magnetic chitosan hydrogel adsorbent was reached after 8 min, this shows that the adsorption process reached the equilibrium. A high adsorption rate was therefore seen at the beginning of the adsorption process as a result of the presence of voids that may trap Pb^2+^ ions. But with time, the spots on the surface of the magnetic chitosan hydrogel adsorbent get saturated, which lowers its effectiveness.

### 3.5 Effect of other metal ions

If an ion alters the adsorption and recovery of Pb^2+^ ions by more than 5%, it is considered to be an interfering ion. Under the established ideal circumstances, the adsorption procedure was conducted with 100 mL of a 40 g/L Pb^2+^ solution and varying concentrations of the interfering ions. The adsorption of the recovered solution was measured after that, and it was compared to the solution that was produced when the sample was recovered without interfering ions. Table 1 shows that the Pb^2+^ ion was retrieved with a 5% error when foreign ions were present and that the measurement was not significantly impacted by the presence of the foreign ions [30].

**TABLE 1:** Effect of other metal ions on the recovery of 40 μg/L lead ions in aqueous samples (0.02 g of adsorbent in pH = 5 for 8 min) (*n* = 3).

Diverse ion	Concentration (μg/L)	Recovery (%)
Zn^2+^	4	96.47 ± 1.45^a^
Ni^2+^	4	98.21 ± 2.98
Ag^+^	4	96.62 ± 1.28
Cd^2+^	4	97.54 ± 2.39
Mn^2+^	4	96.08 ± 2.19

^a^Mean ± standard deviation.

### 3.6 Temperature

**FIGURE 9:**
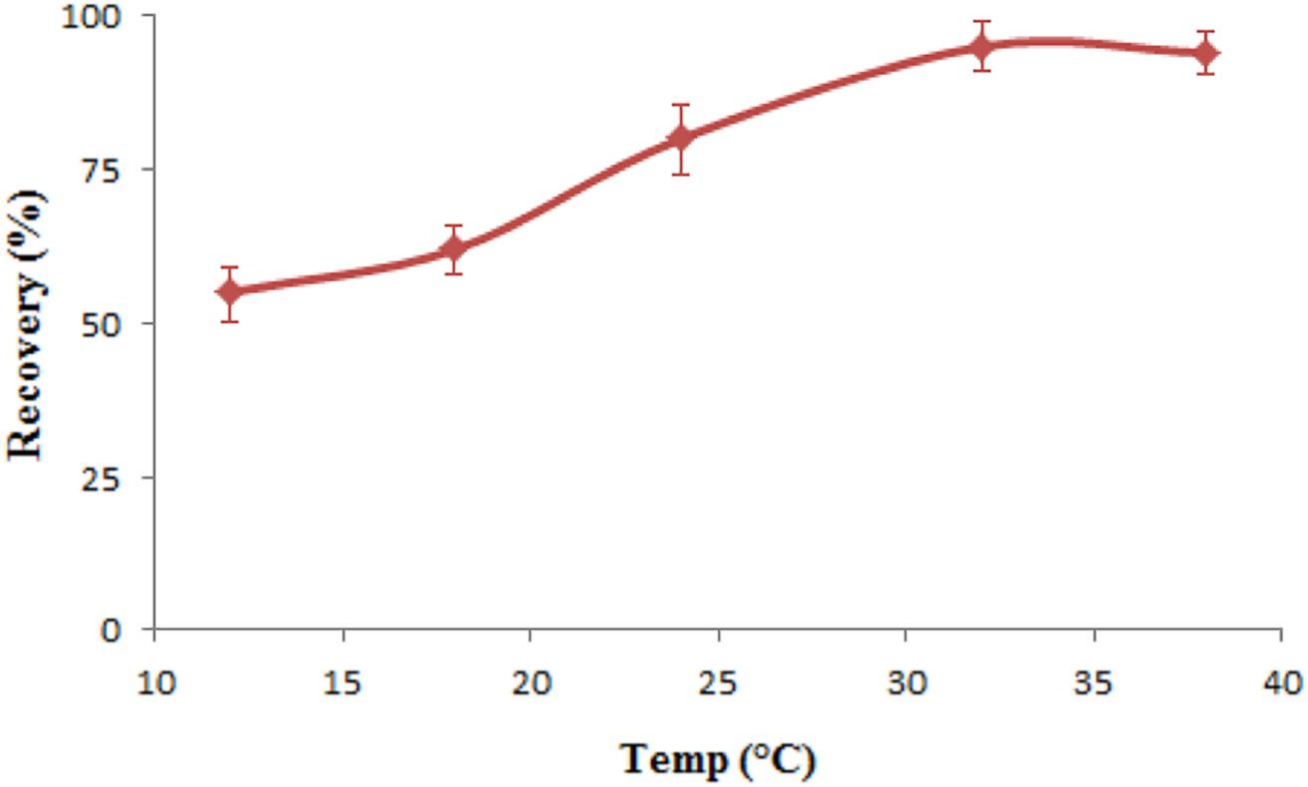
Temperature effect on the adsorption of Pb^2+^ ions (0.02 g of magnetic chitosan hydrogel adsorbent, pH = 5, 8 min).

The best temperatures for Pb^2+^ ions extractions with the refrigerator incubator shaker device were 12, 18, 24, 32, and 38°C. The concentration of the leftover ions was calculated. Investigating the Pb^2+^ ions extractions is the goal of temperature measurement. Figure 9 shows that the efficiency of extraction improved as the temperature rose to 32°C. The activity of the metal ions in the solution.

### 3.7 Salt effect

The salt is still another important characteristic because of the paired ion function between reactants, which improves the interaction between the substances. Regarding the amount of extraction, it is also very important. At 0.10 g, Pb^2+^ ions were shown to be the most effective. The experiment's ideal pH, time, and temperature were used in this section. Salt was used in the Pb^2+^ ions extraction in varying amounts. The Pb^2+^ ions extraction is made possible by adding 0.10 g NaCl to the magnetic chitosan hydrogel adsorbent and Pb^2+^ ions sample, and 1% W/V is the ideal salt concentration for maximal Pb^2+^ ions extraction.

### 3.8 Isotherm study

To further understand the Pb^2+^ ion adsorption process on the surface of the magnetic chitosan hydrogel adsorbent, the gathered data were examined using isothermal models. In this experiment, the Freundlich and Langmuir isothermal models were used. The equivalent energy homogeneous and monolayer adsorption of Pb^2+^ ions throughout the whole magnetic chitosan hydrogel adsorbent surface is the foundation of the Langmuir model in linear and non‐linear form (Equations 2 and 3) [15].

(2) $$\frac{C_{e}}{q_{e}}=\frac{1}{k_{L}q_{\max}}+\frac{C_{e}}{q_{\max}}\;Linear$$

(3) $$q_{e}=\frac{q_{\max}k_{L}C_{e}}{1+C_{e}}\;Non-linear$$

The Pb^2+^ ions concentration at equilibrium is *C*_*e*_ (mg/L), where *q*_*e*_ is the highest amount of Pb^2+^ ions absorption on the magnetic chitosan hydrogel adsorbent (mg/g) and *q*_max_ is the highest amount (mg/g). Where *K*_*L*_ is the Langmuir constant relating to the adsorption's binding energy (L/mg).

In contrast, Equations (4) and (5) states that the multilayer and heterogeneous adsorption processes are the foundation of the Freundlich model in linear and non‐linear form.

(4) $$\ln\;q_{e}=\ln\;K_{F}+\frac{1}{n\;\left(\ln\;C_{e}\right)}\;Linear$$

(5) $$q_{e}=K_{F}\;{C_{e}}^{1/n}\;Non-linear$$

The Freundlich isothermal constants are the adsorption force and capacity, indicated by *n* and *K*_*F*_ (L/g) respectively.

**FIGURE 10:**
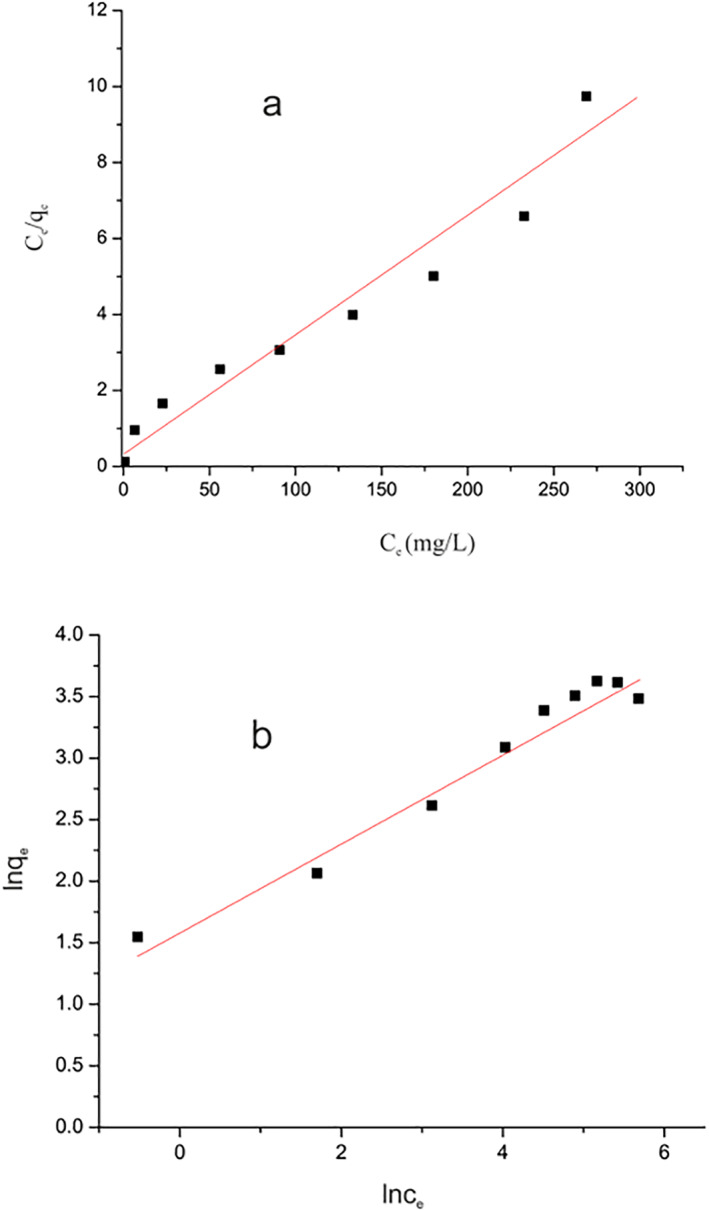
The Langmuir isothermal models for the adsorption of Pb^2+^ (a), and Freundlich isothermal models for the adsorption of Pb^2+^ (b).

As compared to the linear model, the highest amount of Pb^2+^ ions adsorption (*q*_max_) and binding energy (*K*_*L*_) parameters in the current work were shown to be smaller when utilising the non‐linear Langmuir model (Figure 10 and Table 2). The linear Langmuir model (Table 1) was confirmed by respectable linear regression coefficients (*R* ^2^ = 0.95). By comparing the value of the square between the non‐linear model of Freundlich and Langmuir, the lower nominal value of the Freundlich model determines the function model. According to the *R* ^2^ values listed in Table 1, the mechanism of Pb^2+^ ion adsorption on the adsorbent surface is likely multilayer since the adsorption isotherm closely matches the Freundlich model. In addition, 31.74 mg/g of Pb^2+^ ions were shown to be the *q*_max_.

**TABLE 2:** Adsorption of Pb^2+^ ions by magnetic chitosan hydrogel adsorbent isotherm model parameters.

Linear models					
Langmuir isotherm			Freundlich isotherm		
q_max_ (mg/g)	*K*_*L*_ (L/mg)	*R* ^2^	*n*	*K*_*F*_ (L/g)	*R* ^2^
31.74	0.098	0.95	2.76	4.85	0.98

Non‐linear models					
q_max_ (mg/g)	*K*_*L*_ (L/mg)	Chi‐square	*n*	*K*_*F*_ (L/g)	Chi‐square
5.36	0.012	506.14	3.32	6.33	34.25

### 3.9 Lead ions sorption kinetics

To study the kinetic result, the pseudo‐first‐order and second‐order models, two popular kinetic models of adsorption, were utilised (Figure 8). The pseudo‐first‐order pattern in the linear and non‐linear model is represented by the equations that follow (Equations 6 and 7):

(6) $$\log\left(q_{e}-q_{t}\right)=\log\;q_{e}-\frac{k_{1}t}{2.303}\;linear$$

(7) $$q_{t}=q_{e}\left(1-e^{-kt}\right)\;non-linear$$

The *q*_*e*_ and *q*_*t*_ components in Equation indicate the equilibrium adsorption (mg/g) value at *t* (min), whereas *k*_1_ (1/min) represents the pseudo‐first‐order constant in the rate model. The linear and non‐linear model of the pseudo‐second‐order pattern might be expressed as follows:

(8) $$\frac{t}{q_{t}}=\frac{1}{k_{2}q_{e}^{2}}+\frac{t}{q_{e}}\;linear$$

(9) $$q_{t}=\frac{k_{2}q_{e}^{2}t}{1+k_{2}q_{e}t}\;non-linear$$

The *k*_2_ (g/mg min) is known as the rate constant in Equation (8).

**FIGURE 11:**
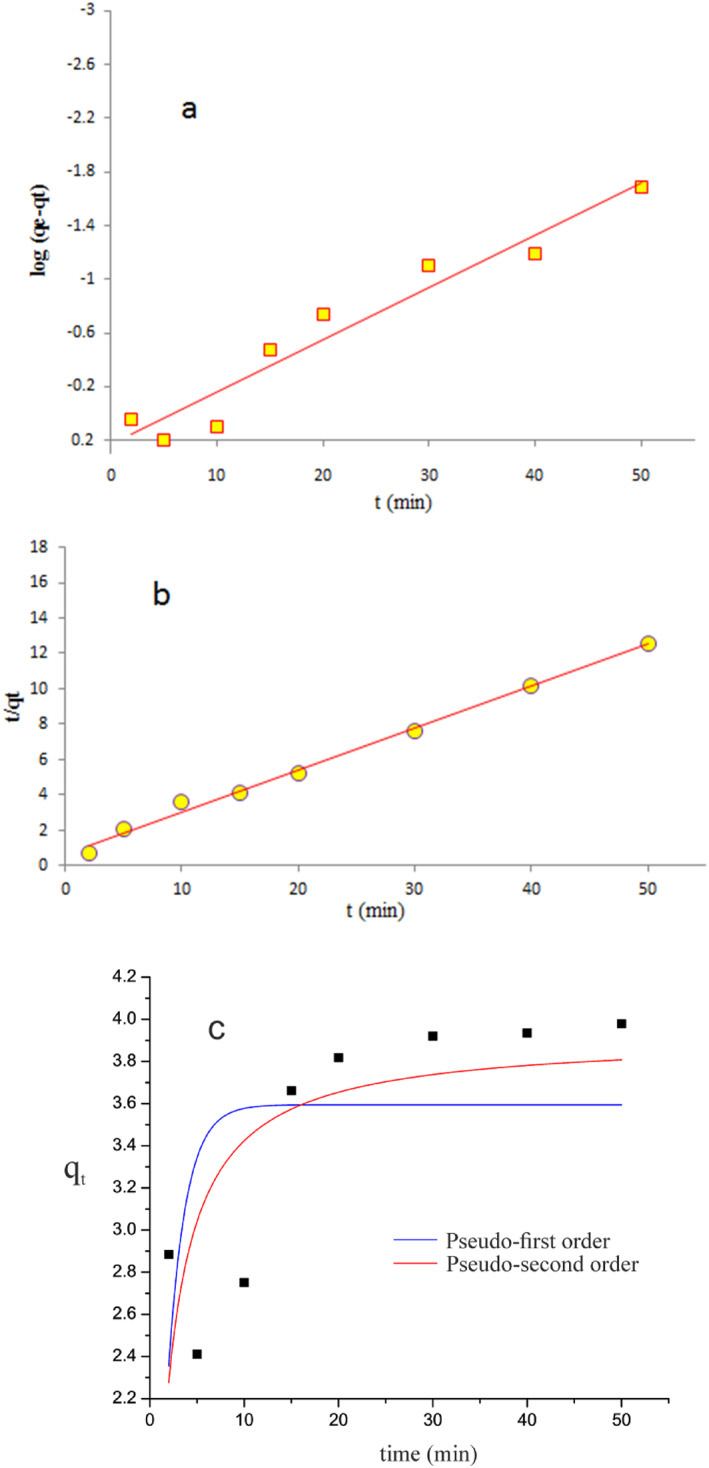
The pseudo‐first‐order linear (a), pseudo‐second‐order linear (b) and non‐linear (c) models of Pb(II) adsorption kinetics.

The non‐linear forms of pseudo‐first‐order and pseudo‐second‐order kinetic models for the adsorption of Pb(II) on magnetic acrylamide nanocomposite are represented in Figure 9. The determined constants and theoretically derived *q*_*e*_ are shown in Table 2 as a result of graphing *q*_*t*_ versus *t*. To calculate the kinetic parameters for the adsorption of Pb(II) ions on magnetic acrylamide nanocomposite, the pseudo‐second‐order model appears to be more plausible than the pseudo‐first‐order model, according to computed values of the Chi‐square test analysis for non‐linear models of pseudo‐first‐order and pseudo‐second‐order, which were found to be 0.38 and 0.22 respectively. According to the correlation coefficient factor value, the pseudo‐second‐order linear pattern of Pb(II) adsorption fits the kinetic result more closely than the pseudo‐first‐order linear adsorption pattern (Figure 11). The kinetic constant values for Pb(II) ions in the pseudo‐second‐order linear equation are listed in Table 3. According to the *R* ^2^ value, the pseudo‐second‐order linear model is more adapted to explain the Pb(II) sorption onto the magnetic acrylamide nanocomposite than a model of the pseudo‐first‐order. Chemical adsorption is the rate‐limiting stage in the adsorption process.

**TABLE 3:** Estimated parameters of adsorption kinetics for the Pb(II) sorption on magnetic acrylamide nanocomposite.

Pseudo‐first order model	Pseudo‐second order model		
Linear form			
*q*_*e*_ (mg/g)	0.58	*q*_*e*_ (mg/g)	4.20
*k*_1_ (1/min)	0.09	*k*_2_ (g/mg min)	0.08
*R* ^2^	0.94	*R* ^2^	0.99
Non‐linear form			
*q*_*e*_ (mg/g)	3.59	*q*_*e*_ (mg/g)	3.92
*k*_1_ (1/min)	0.53	*k*_2_ (g/mg min)	0.18
Chi‐square	0.38	Chi‐square	0.22

### 3.10 Analytical procedure

This approach has an outstanding linear range of 0.5–26 mg/L for Pb^2+^ ions. The limits of detection (LODs) for Pb^2+^ ions adsorption by magnetic chitosan hydrogel adsorbent were reached by utilising a signal‐to‐noise ratio of 3 (LOD = 2.12 μg/L). These findings show that magnetic chitosan hydrogel adsorbent is an effective adsorbent for extracting Pb(II) from different sample waters.

## 4 DESORPTION STUDY

**FIGURE 12:**
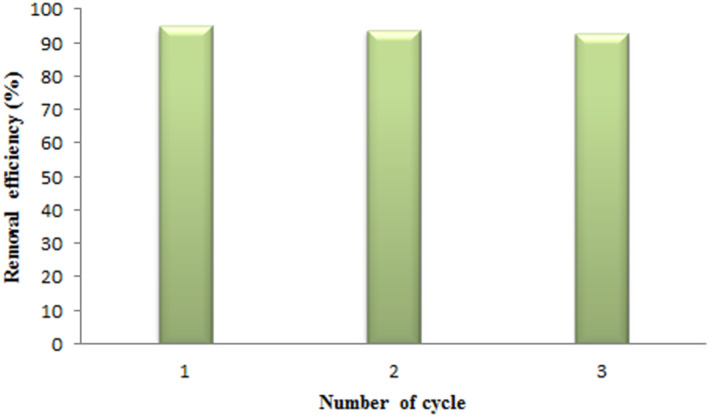
Reusability of magnetic acrylamide nanocomposite.

After using the desorption‐adsorption cycle, the synthetic adsorbent must be regenerable, which is both economically significant and advantageous. This section performed adsorption–desorption experiments of Pb^2+^ ions by the magnetic acrylamide nanocomposite under ideal circumstances in three consecutive cycles to assess the viability of reusing the adsorbent (Figure 12). The adsorption percentage stayed upper 90% for the three cycles. As a result, the synthetic magnetic acrylamide nanocomposite is effective in removing Pb^2+^ ions from the aqueous solution and may be employed up to three more times.

### 4.1 Measurement of Pb^2+^ ions in real water samples

To test the applicability of the presented method for measuring Pb^2+^ ions in actual samples, the concentration of Pb^2+^ ions was measured by 0.02 g of adsorbent in pH = 5 for 8 min in different aqueous samples with a volume of 200 mL. Regarding this, the water samples, such as tap water (collected in Varamin on April 15, 2022, after running the water for 10 min), well water (taken in Tehran on April 22, 2022), and industrial wastewater (taken in Charmshahr on March 18, 2022) (Table 4). The measuring of the lead ions concentration using the graphite furnace atomic spectrometer.

**TABLE 4:** Pb^2+^ ions determination in actual water samples (number of repetitions 3 times).

Samples	Spiked (μg/L)	Found (μg/L)	Relative recovery (%)
Tap water	0.0	ND	‐
	5.0	5.03 ± 1.16^a^	97.22 ± 2.12
	15.0	20.43 ± 1.85	98.32 ± 1.87
Well water	0.0	6.35 ± 1.57^a^	98.72 ± 1.21
	5.0	12.12 ± 1.92	97.42 ± 2.04
	15.0	22.64 ± 1.46	98.82 ± 1.61
Industrial wastewater	0.0	6.87 ± 2.24	98.36 ± 2.85
	5.0	12.20 ± 2.57	97.71 ± 2.93
	15.0	22.95 ± 2.68	97.02 ± 2.41

Abbreviation: ND, not detected.

^a^ Mean ± standard deviation.

### 4.2 Comparison of this technique with another method

The employed methodology is superior to the approaches because it has a high Pb^2+^ ion loading, performs extraction near neutral pH, and has a low equilibrium time. Magnetic chitosan hydrogel adsorbent may be easily separated using a magnet instead of centrifuges or filtrations. Additionally, the use of pricey and dangerous organic solvents is decreased when using the magnetic chitosan hydrogel adsorbent with extraction in an aqueous sample (Table 5) [11, 12, 17, 31].

**TABLE 5:** Comparison of this adsorbent with another method for Pb^2+^ ions extraction.

Approach	*q_m_* (mg/g)	pH	Equilibrium time	Reference
Magnetic chitosan hydrogel adsorbent	31.74	5	8 min	This work
Amino functionalised magnetic graphenes composite	27.95	6	200 min	[11]
Magnetic biochar composite	26.08	5	120 min	[12]
Magnetised and non‐magnetised biochar from douglas fir	25.29	5	180 min	[31]
Bimetallic coordination polymer adsorbent	175	4	30 min	[17]

## 5 CONCLUSION

To increase the extraction properties and Pb^2+^ ions adsorption in the current study, a magnetic chitosan hydrogel adsorbent was employed to effective extraction heavy metal ions from water samples. The structure of magnetic chitosan hydrogel adsorbent was then examined using an analytical approach, and different amounts of magnetic chitosan hydrogel adsorbent were used to effectively extract Pb^2+^ ions from a variety of water samples. The *q*_max_ for Pb^2+^ ions was 31.74 mg/g at pH = 5. According to the adsorption isotherms, the Freundlich model was the most useful one to explain the sorption of Pb^2+^ ions by the magnetic chitosan hydrogel adsorbent. According to the *R* ^2^ = 0.99, the pseudo‐second‐order model is more suited to explain the Pb(II) sorption onto the magnetic chitosan hydrogel adsorbent. In the conclusion, the recommended approach offers an effective way to measure trace levels of lead ions in a variety of water samples. Notable features include high efficiency, rapid separation rate, high adsorption capacity, and good recovery. The magnetic chitosan hydrogel adsorbent may be used up to three more times since it works well at removing Pb^2+^ ions from aqueous solutions.

## AUTHOR CONTRIBUTIONS

**Arman Samadzadeh Mamaghani**: Investigation; Resources; Software. **Mohammad Hojjati**: Validation; Visualisation; Writing – original draft.

## CONFLICT OF INTEREST STATEMENT

The authors declare that they have no known competing financial interests or personal relationships that could have appeared to influence the work reported in this paper.

## Data Availability

Data sharing not applicable to this article as no datasets were generated or analysed during the current study.
